# Role of T cells in cervical cancer

**DOI:** 10.6026/97320630019556

**Published:** 2023-05-31

**Authors:** Issam Alshami, Raghad O Alattas, Alofi Waad A, Sayed Anwar A

**Affiliations:** 1 Department of Medical Microbiology and Immunology, Taibah University, Madinah, Saudi Arabia; 2 College of Medicine, Taibah University, Madinah, Saudi Arabia

**Keywords:** Cervical cancer, Human papillomavirus, T cells

## Abstract

Cervical cancer is an important health problem and it is considered the fourth most lethal women's cancer worldwide. The intertumoral T cell pool is exposed in a number of immunosuppressive pathways. Therefore, it is of interest to document the effect of cervical cancer on immune system, the role of T cells in the development and pathogenesis of cervical cancer. HPV is considered the most important risk factors for developing cervical cancer, HPV 16 and 18, the two most common oncogenic types which are high risk HPV cause 70% of cervical cancer cases. In the cervical mucosa, the proportion of CD4+ and CD8+ T cells is related to the severity of the lesions. Cervical cancer can be treated by immunotherapeutic vaccine which involves T cells. T cells play an important part in cervical cancer pathogenesis because HPV exploits several methods to avoid host T-cell immune surveillance. T-cell-based immunotherapy is important because it is selective and has therapeutic potential.

## Background:

Cervical cancer (CC) is an important public health problem and is considered the fourth most lethal women's cancer worldwide. Studies indicated that in 2012, about 527,600 new CC cases were detected and around 265,700 deaths globally [1] . There are 10.3 million women in Saudi Arabia aged 15 and up who are at risk of acquiring CC. According to current statistics, 358 women are diagnosed with CC per year, with 179 dying from the disease.CC is the eighth most common disease among Saudi Arabian women, and the eighth most common cancer among women aged 15 to 44 [2]. The majority of instances occur in developing nations where there are no adequate screening measures in place. The majority of women with early-stage tumors can be treated, while treatment-related long-term morbidity is prevalent. Randomized clinical trials have demonstrated that chemoradiotherapy should be considered the standard of care for women with locally advanced malignancies; nevertheless, the treatment's applicability to women in less industrialized nations is mainly unknown [3]. CC is driven by a variety of factors like smoking and immune system dysfunction. Human Papilloma Virus (HPV) infection most important risk factor also it considered the most common sexually transmitted viral infection around the world [3] [4]. Studies referred that the presence of HPV is not enough to cause the disease, but there is a need for the presence of genetic factors [5] . HPV is double stranded DNA viruses it encodes eight major proteins, six located in the early (E) regions (E1, E2, E4, E5, E6, and E7) and two located in the late (L) regions (L1 and L2). E5, E6, and E7 are viral oncogenes which lead to transformation of the cells; also E6 and E7 lead to inactivate cellular tumor suppressor proteins which are p53 and pRb. In addition, these oncogenes abolish cell cycle regulation, apoptosis, and DNA repair which enhance the development of cancer [6]. Until now, there are over 200 types of HPV have been discovered according to the degree of oncogenic capacity. HPV 6, 11, 40, 42, 43, 44, 53, 54, 61, 72, 73, and 81 called "low- risk" HPV (lrHPV) because they do not cause malignant transformation [6]. While, HPV 16, 18, 31, 33, 35, 39, 45, 51, 52, 56, 58, 59, and 68 called "high-risk" HPV (hrHPV) because they can cause malignant transformation, this type has a strong relation with CC [4] . Migration of some cells occurs such as T lymphocytes and Langerhans cells, to the squamous epidermis due to their response to high-risk hrHPV infection, the responsible for the regression or progress of these cancerous lesions that associated with HPV such as CC is dependent on the interaction of different cellular immune. Therefore, understanding the role of T cells in CC is very important it play a major role in the progression of CC by its relation with HPV at the same time, it has a role in the treatment of CC by different ways [6]. Therefore, it is of interest to document the effect of CC on the Immune system, identify the role of T cells in the development and pathogenesis of CC and demonstrate the role of T cells in the treatment of CC. Infection with the hrHPV remains a significant beginning event in CC carcinogenesis and one of the most important risk factors for developing CC in individuals with the disease [7]. Recent evidence from murine and human cancer studies suggests that intratumorally T cells exhibit a wide range of dysfunctional states, determined by the complex inhibitory signals present in the tumor microenvironment. We cover the current state of knowledge about T cell dysfunction in cancer, the value of novel technologies for dissecting such failure at the single-cell level, and how our growing knowledge of T cell dysfunction might be used to design individualized anticancer strategies. Because the intra tumoral T cell pool is exposed to a variety of immunosuppressive pathways, CC patients may have a wide range of dysfunctional cells [8].

## The Immune System and Cervical Cancer:

Immune system deficiencies play a crucial influence in CC progression. Also, HPV infection is thought to cause a mainly cell-mediated immune response, and evidence of T helper cell participation in regressing lesions has been found. According to one study, women who were free of HPV had more Langerhans cells [8]. Tumors are identified by the immune system, and through a process known as immunosurveillance, they can be treated or prevented. Mucosal immunity is the first line of defense in the human body; cellular and humoral immunity also play important roles in carcinogenesis and disease progression. Also, the immune system is closely linked to the hallmarks of precancer High and low-grade squamous intraepithelial lesion progression. Also, T cells, B cells, Dendritic cells (DCs), Natural Killer cells (NK), and macrophages are among the immune cells that make up the tumor microenvironment. Immune cells are classified as either immunoactivity or immunosuppressive depending on their function. The interaction between Antigen-presenting cells (APCs) and T lymphocytes controls the immune response to tumor cells. APCs have a number of costimulatory and corepressor molecules on their membranes that bind to receptors on antigen-presenting T-cell membranes, these molecules are known as immune checkpoints which play a role in charge of restoring homeostasis following an immune response. One of the hallmarks of HPV infection persistence is the virus' ability to avoid efficient immune system identification [2]. HPV is able to avoid the immune response despite many immune defensive mechanisms. Because virus location during early phases [i.e., confined to basal epithelial cells) and HPV protein production tends to be limited during this initial phase, the immune response to HPV is often modest. HPV16 and 18 also appear to dampen a cellular immune downregulating the expression of antigen-presenting pathway components, the inflammasome, antiviral production, pro-inflammatory and chemotactic cytokines, and components downstream of activated pathogen receptors [15]. Infected keratinocytes' innate immune signaling pathways are suppressed by HPV proteins also HPV protein can downregulate the processing of classical MHC molecules to the cell surface, resulting in the avoidance of CD8 + detection of infected cells, which targets the adaptive immune response [1]. Furthermore, the presence of a large number of tumors infiltrating lymphocytes is linked to the advancement of CC illness [8].

## The role of T cells in the development of CC:

The importance of T cells in cervical cancer development can be determined by the fact that HPV uses several strategies to avoid host T-cell surveillance, allowing infection to persist and cancer to spread. HPV-specific CD4 T cells recovered from lymph node biopsies of cervical cancer patients have also been shown to decrease responder T cell growth and production of IFN-γ and interleukin-2 (IL-2) [6]. Treg cells are an immunosuppressive subgroup of T cells that performs this important job while also having negative effects on tumor immunosurveillance and anti-tumor immunity. Evidence from cancer patients suggests that elevated Target activity is linked to inadequate immunological responses to tumor antigens, potentially contributing to immune dysfunction. An immune response or suppression will result from an imbalance among these T cells. The balance between Treg cells and Th17 cells is thought to modulate the immune response and is an important element in regulating helper T cell function in autoimmune disorders and graft versus host disease [9]. Previous research has shown that Th1/Th2 cell imbalances and Th17/Treg cell imbalances in CC or CIN patients determine the link between immunological imbalances. In addition, the situation in CC patients was more serious than in CIN patients [9]. According to the studies, the more severe the disease was, the more visible the changes in the four categories of CD4 + T cells were. The cell percentages were all connected to the stage of the tumor, the extent of tumor vaso invasion, and the presence of lymph node metastases. The Th1/Th2 ratio fell as the disease progressed, as Th1 reduced and Th2 grew rapidly. Despite Treg cell overexpression, the Th17/Treg ratio increased due to the significant increase in Th17. Increased levels of Tregs were also seen in the cervical tumor site and in the lymph nodes of cervical cancer patients, according to some reports an increase in Treg cells was linked to a state of immunosuppression [9]. Emerging evidence suggests that in the case of HPV, Th1 cells, which generate IFN- and lymphotoxin, are essential for disease resistance, whereas Th2 cells, which produce IL-4, IL-5, and IL-13, aid in the propagation of the infection. This idea was bolstered by the fact that Tumor infiltrating lymphocytes (TILs) in cervical cancers are predominantly Th2/Tc2 polarized, and the draining nodes have a higher proportion of Treg cells, which are recruited to suppress the antitumor immune response because Treg cells may interfere with both the induction and the effector response. Even cytokines linked with Th17/Treg cells play a role in the onset and progression of cervical cancer. In a word, HPV has a variety of techniques for avoiding the immune system of the host [6].

## The role of CD4+ and CD8+ T-cell in the pathogenesis of CC:

Tumors contain a range of cell membrane bound antigens that the immune system recognizes as non-self, triggering a cytotoxic immune response defined by the infiltration of CD4, CD8, antigen-presenting cells, and other lymphoid components [10]. The inhibition of CD4+ and CD8+ T-cell responses to the HPV is linked to the development of CC. Rather than spreading to distant organs, the disease mostly affects lymph nodes, vagina, bladder and pelvis [11]. For the role of CD8+T-cell responses to E6 and E7 using enzyme-linked immunospot assays in persons with incident or prevalent HPV 16 or 18 infections found no significant differences in the frequency of positive. In addition, in patients with significant early-stage cervical carcinoma, a high number of intraepithelial CD8+ tumor-infiltrating lymphocytes has been linked to the absence of lymph node metastases. These data suggest that the failure of HPV-specific T-cell responses is linked to the development of(hrHPV)-positive cervical cancer [12]. The proportion of CD4+ and CD8+ T lymphocytes in the cervical mucosa is proportional to the severity of the lesions. Individuals with Cervical intraepithelial neoplasia (CIN) in remission or low-risk HPV-induced genital warts had a higher proportion of CD4+ T cells than CD8+ T cells, whereas patients with advanced lesions and invasive CC have a higher proportion of CD8+ T cells. Despite the fact that the number of cells responsible for tumor cell eradication has increased, they are anergic and nonfunctional [11]. HPV has the ability to bypass the innate immune system and enter epithelial cells. DCs absorb the HPV antigen as soon as they enter the cell and go through a maturation process. The antigen is subsequently sent to Major Histocompatibility Complex MHC class I or II molecules on the cell surface by the phagolysosome. The CD4+andT cells will bind to the T-cell receptor once they have bound to the CD4+and T cells [13].

## The effect of HLA-I on CD4 and CD8 in the pathogenesis of CC:

**Figure 1:**
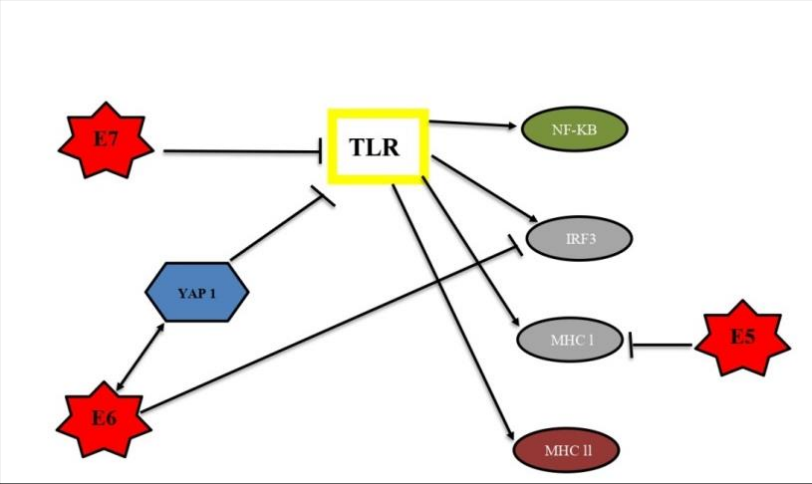
HPV infection triggers a variety of mechanisms. The host immune system responds to HPV infection by activating toll-like receptors [TLRs], which then activate nuclear factor-kappa B [NF-kB] and interferon regulatory factor 3 [IRF3], which then activate pro-inflammatory and antiviral cytokines. The major histocompatibility complex [MHC] class I and II are also activated by TLR. The HPV, on the other hand, can use its viral oncoprotein E5 to block MHC class I processes. The oncoprotein E6 has the ability to stop IRF3 from being produced. E6 binds to Yes-associated protein [YAP1], preventing it from being degraded and blocking TLR signaling.

CD8 and CD4 molecules, as well as human leukocyte antigen-I(HLA-I), are critical components of the human immune system. The main function of HLA-I is to convey viral antigens to immune cells such T cells, which then activate antigen-specific Cytotoxic T lymphocytes (CTLs) in the human body. Low or absent expression of such molecules is likely to play a key role in tumor cell immune evasion [14]. HLA class I loss is common in CC and is caused mostly by genetic abnormalities in the 6p21.3 locus, the non-classical HLA-G appears to be one of the most effective molecules for suppressing the innate and/or adaptive immune response by diverse immune system pathways, among other techniques adopted by tumor cells to escape identification by different immune effectors [13]. HLA-G has been shown to suppress CD8+ T cell cytotoxicity, as well as NK cell cytotoxicity and T-cell alloproliferation also several studies have found that HLA-G expression in cancer patients relates to tumor growth and may be a therapeutic target, it has also the potential to modify and change cytokine production from a T-helper Th1 to a Th2 profile [13]. 70, on the other hand, are linked to cervical cancer lesions and Interferon regulatory factors (IRF). Changes in immune cells in the immunological response to hrHPV are the cornerstone for the transition from hrHPV infection to CC is a compromised adaptive immune system. In CIN and carcinogenesis, distinct immune cell profiles correspond to different stages of disease progression. The immune system is adapted by hrHPV infection to produce a favorable milieu for persistent infection and lesion progression, thanks to the changes and modifications induced by the virus T cell activation is harmed by HPV infection [16]. According to a study, the expression of the Toll-like receptor-9 (TLR-9) gene varies depending on the stage of cervical cancer development. The TLR-9 gene was found to have lower expression in CIN 1 than in CIN 2/3 and to have the highest expression in squamous cell carcinoma samples. Continuous overexpression of E6 and E7 oncoproteins, on the other hand, may downregulate TLR-9, impairing the subsequent interferon response, resulting in immune evasion and prolonged infection. The HPV E7 oncoprotein has been shown to bind to Histone deacetylase 1 and block histone acetylation, hence disrupting TLR9 signaling. HPV up regulates epidermal growth factor receptor expression, causing interferon-related developmental regulator 1 expression to decrease cytokine production by reducing NF-B, according to another study. According to a recent study, the Hippo-Yap pathway is involved in the development of cervical cancer. The Yes-associated protein (YAP1), a key activator of the Hippo signaling system, interacts with the HPV E6 oncoprotein to initiate and enhance cervical cancer progression. The HPV oncoprotein binds to YAP1 and prevents its degradation in a synergistic manner. The oncogene YAP1 was found to be amplified in human squamous cervical malignancies, and its overexpression in cervical epithelial cells caused squamous cell carcinoma to grow in a mouse model. TLR 2 and 4, which are important components of innate immunity, are downregulated when YAP1 is upregulated, according to the study. Although YAP1 could be a possible predictive biomarker in cervical cancer, the particular pathways linked with YAP1-induced cervical cancer are currently being explored. In summary, HPV-associated cervical cancer occurs when the virus evades the immune system of the host, resulting in additional cellular dysfunction (Figure 1) [15].

## The role of HPV and cytokines in CC progression:

Cytokines are local immune mediators that recruit and govern the function of immune cells to control HPV infection. HPV causes the host immune system to become more tolerant of infection, allowing it to persist and advance CIN. High-risk HPVs are linked to mucosal infection, while low-risk HPVs are linked to cutaneous lesions. hrHPVs 18, 31, 33, 34, 35, 39, 45, 51, 52, 56, 58, 59, 66, 68, and 70, on the other hand, are linked to cervical cancer lesions and Interferon regulatory factors (IRF). Changes in immune cells in the immunological response to hrHPV are the cornerstone for the transition from hrHPV infection to CC is a compromised adaptive immune system. In CIN and carcinogenesis, distinct immune cell profiles correspond to different stages of disease progression. The immune system is adapted by hrHPV infection to produce a favorable milieu for persistent infection and lesion progression, thanks to the changes and modifications induced by the virus T cell activation is harmed by HPV infection [16]. According to a study, the expression of the Toll-like receptor-9 (TLR-9) gene varies depending on the stage of cervical cancer development. The TLR-9 gene was found to have lower expression in CIN 1 than in CIN 2/3 and to have the highest expression in squamous cell carcinoma samples. Continuous overexpression of E6 and E7 oncoproteins, on the other hand, may downregulate TLR-9, impairing the subsequent interferon response, resulting in immune evasion and prolonged infection. The HPV E7 oncoprotein has been shown to bind to Histone deacetylase 1 and block histone acetylation, hence disrupting TLR9 signaling. HPV up regulates epidermal growth factor receptor expression, causing interferon-related developmental regulator 1 expression to decrease cytokine production by reducing NF-B, according to another study. According to a recent study, the Hippo-Yap pathway is involved in the development of cervical cancer. The Yes-associated protein (YAP1), a key activator of the Hippo signaling system, interacts with the HPV E6 oncoprotein to initiate and enhance cervical cancer progression. The HPV oncoprotein binds to YAP1 and prevents its degradation in a synergistic manner. The oncogene YAP1 was found to be amplified in human squamous cervical malignancies, and its overexpression in cervical epithelial cells caused squamous cell carcinoma to grow in a mouse model. TLR 2 and 4, which are important components of innate immunity, are downregulated when YAP1 is upregulated, according to the study. Although YAP1 could be a possible predictive biomarker in cervical cancer, the particular pathways linked with YAP1-induced cervical cancer are currently being explored. In summary, HPV-associated cervical cancer occurs when the virus evades the immune system of the host, resulting in additional cellular dysfunction (Figure 1) [15].

**Figure 2:**
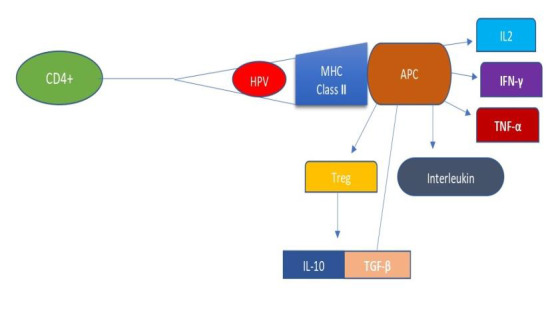
MHC class II-related mechanisms during HPV infection. When HPV infects the epithelium, the host immune system recognizes the antigen and initiates phagocytosis. The antigen is sent by the phagolysosome to attach to the MHC class II molecule. The antigen-presenting cell [APC] then activates CD4+ and T lymphocytes, causing them to become cytotoxic. Interferon gamma [IFN-y], tumor necrosis factor alpha [TNF-a], and interleukin 2 are pro-inflammatory and antiviral cytokines that are activated when APC is activated [IL-2]. Also stimulated are the interleukins. The generation of regulatory T lymphocytes is also triggered by the activation of antigen presentation cells [APC] [Tregs]. Interleukin 10 [IL-10] and transforming growth factor beta [TGF-ß] will be activated by Tregs, inhibiting APC function.

HPV has the ability to get past the innate immune system and enter epithelial cells. DCs absorb the HPV antigen as soon as they enter the cell and mature. The antigen is then delivered to MHC class I or II molecules on the cell surface by the phagolysosome. When CD4+ and T cells bind to the T-cell receptor, they will also bind to it. Antigen-presenting cells (APC) will then activate CD4+ and T lymphocytes, causing them to produce cytotoxicity. The pro-inflammatory and antiviral cytokines Interferon gamma (IFN-γ) and Tumor necrosis factor alpha (TNF-α) are both activated when APC is activated. This causes macrophage stimulation, which promotes inflammation or tumor immunity. Interleukins are also activated in response to infections outside the cell. The activation of APC, on the other hand, causes the creation of Tregs. IL-10 and transforming growth factor beta (TGF-δ[will be activated by Tregs, inhibiting APC action. As a result, the amount of Treg cells produced correlates with the transition of cells from normal to precancerous lesions and cancer in HPV cancer progression. Women with persistent HPV 16 infection have been found to have considerably more Tregs than women who are HPV-negative. Furthermore, Treg-inducing substances including TGF-δ1 were found to be elevated in lesions moving from CIN 1 to invasive cervical cancer in another investigation (Figure 2) [16].

## The role of T cells in the treatment of CC:

## Immune therapeutic vaccines:

**Figure 3:**
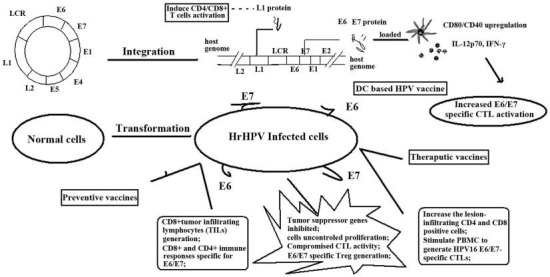
Vaccine mechanisms in the prevention and treatment of HPV infection and the progression of CIN. Vaccines aim to increase HPV oncoprotein-specific immune responses, such as cytokine generation, CTL initiation, and immune system remodeling, helping to treat HPV infection, CIN, and CC at all stages. CD stands for cluster of differentiation; IL stands for interleukin; IFN stands for interferon; DC stands for dendritic cell; hrHPV stands for high-risk human papillomavirus; CTL stands for cytotoxic T cell; PBMC stands for peripheral blood mononuclear cell; Treg stands for regulatory T cell; LCR stands for long control region.

In the fight against HPV infection, the immune system is crucial; the course of disease in HPV-induced carcinogenesis will be determined by changes in the microenvironment and the interaction between virally infected keratinocytes and the local immune milieu. By selectively recognizing virus-associated tumor cells or unleashing negative feedback on CTLs , which allows them to target neoplastic cells, also the vast majority of clinical trials for HPV-targeted treatments rely on vaccines to trigger cell-mediated immune responses. The technique for treating hrHPV infection and CC is to find a target to create a specific immune response. Vaccination against HPV16 and 18 is now routinely utilized as a preventative measure. One of the most successful ways for eliciting immune responses against hrHPV is antigen specific immunotherapy [18]. Due to its propensity to create CD8+ tumor infiltrating lymphocytes, Listeria monocytogenes (LM) has been employed to remove visible, vascularized tumors in multiple mouse models. A truncated Listeriolysin O (LLO) attached to E7 and a fragment of the ActA protein fused to E7, respectively, have been used to generate two vaccines, LM-LLO-E7 and LM-ActA-E7. These vaccinations break central tolerance by increasing the number of low avidity CD8+ T cells that are specific for E7. HPV16 E7-HBcAg-Hsp65 (VR111), a new fusion protein that could elicit an E7-specific CD8+ T cell response, is a novel possible preventive vaccination ]. Therapeutic vaccinations are primarily focused at oncoproteins E6 and E7, the only viral proteins expressed in CC and precursor lesions, with the goal of clearing hrHPV infections and hrHPV-related cervical lesions. Previously, therapeutic vaccines targeting E6/E7 oncoproteins were employed to enhance the number of lesion-infiltrating CD4+ and CD8+ T cells in intraepithelial neoplasia lesions. A novel HPV16 E6 and E7 gene plasmid containing Oligo-mannose liposomes (OML-HPV) was created in a trial for CC immunotherapy. HPV16 E6 specific CTLs could be produced from peripheral blood mononuclear cells for HPV16+ CC patients using OML-HPV stimulation [16]. The DC-based HPV vaccine was discovered to be a promising tool for preventing and treating hrHPV infection, as well as CC, during the analysis of HPV vaccinations. DCs derived from peripheral blood monocytes treated with IL-4 and granulocyte-macrophage colony-stimulating factor were treated with HPV16 mE7, resulting in enhanced expression of co-stimulatory molecules CD80 and CD40, as well as increased production of IL-12p70 and IFN-. In individuals with hrHPV-associated CC, this fraction of DCs could upregulate E7-specific CD8+ T cell responses. A DC-based HPV vaccination is HPV16 E7 polypeptide. The immunotherapeutic activity of DC loaded with hrHPV16 E7 polypeptide in conjunction with CpG-ODN2006 was also notable. When Severe combined immunodeficiency [SCID) mice are given antigen-loaded DCs, their tumors shrink and their IgG and IFN levels rise. When SCID mice are injected with antigen-loaded DCs, tumor growth is reduced, IgG and IFN- levels are increased, and CTL activity is increased. The HPV-16 E6/E7 fusion protein activated distinct protective immune capabilities, resulting in an efficient approach against CC cell proliferation (Figure 3) [17].

## Adoptive Immunotherapy:

The T-cell vaccination serves as both a preventative and a therapeutic tool; these vaccines are intended to stimulate the patient's HPV-specific CTLs. A different strategy known as "adoptive immunotherapy" recreates the immunization process in the laboratory to stimulate and generate huge numbers of CTLs, the inclusion of cytokines can influence the activation and multiplication of CTLs in the lab, and the capacity to remove these T cells from the host, who may be harbouring immune-suppressive substances produced by CC, is another benefit over the vaccine strategy. CTLs can be re-infused into patients in large quantities to overwhelm tumors with tumor-specific killer cells, this method is expected to result in a more than 10-fold larger induction of T-cells than can be achieved by vaccination the patient. Exogenous T cell injection would allow this natural limit to be surpassed in the hopes of achieving more effective tumor management, T-cells are programmed to die after a given degree of stimulation, therefore CTL genetic engineering may be required to bypass the immune system's natural regulatory systems. CTL treatment aims to develop autoimmune disease that only attacks HPV-infected cells in the body in this way. If T-cell insufficiency contributes to the rapid advancement of CC in AIDS patients, T-cell augmentation through vaccination or adoptive immunotherapy may improve disease control in otherwise healthy patients with CC. Despite the fact that in vitro vaccination follows the same basic immunologic principles as in vivo vaccination, modern laboratory immunology techniques and settings may be able to overcome vaccination barriers that arise within the affected host [18] . Also, significant number of tumor-specific cytotoxic T cells is injected into cancer patients with adoptive T-cell therapy with the purpose of detecting, targeting, and eliminating tumor cells. T-cell immunotherapy has been proven to eliminate solid tumors using adoptive transfer of in vitro chosen TILs in preliminary studies. Patients with metastatic CC who had previously had platinum-based chemotherapy or chemo radiation were treated with a single infusion of tumor-infiltrating T-cells, chosen for HPV E6 and E7 reactivity HPV-TILs in a sentinel trial [19].

## Conclusion:

The immune system is linked to the high and low grade squamous intraepithelial lesion progression markers of precancer. The immune response to tumor cells is controlled by the interaction between APCs and T lymphocytes. HPV uses numerous ways to elude host T-cell surveillance, allowing infection to persist and cancer to grow, demonstrates the role of T cells in CC development. In the progression of CC, HPV and cytokines play a key role. When HPV infects the epithelium, the antigen is recognized by the host immune system, which triggers phagocytosis and subsequently several cytokines are activated. T cells can help in the treatment in CC, in preliminary trials adoptive transfer of TILs was shown to remove solid tumors using T-cell immunotherapy. CTL treatment attempts to establish an autoimmune illness that selectively affects HPV-infected cells in the body.

## References

[1] Lee SJ (2016). J Gynecol Oncol..

[2] Sayed AA (2021). Saudi Med J..

[3] Panici PB (2004). Curr Ther Nov Approaches..

[4] Van D (2014). Press Medicale..

[5] Fang J (2014). Tumor Biol..

[6] Chauhan SR (2018). Curr Probl Cancer..

[7] Kamal M (2021). Br J Cancer..

[8] Thommen DS, Schumacher TN (2018). Cancer Cell..

[9] Lin W (2020). BMC Womens Health..

[10] Balasubramaniam SD (2019). Med..

[11] Heeren AM (2019). J Immunother Cancer..

[12] Garbuglia AR (2020). Front immunol..

[13] Maskey N (2019). Cancer Manag Res..

[14] Fan JT (2011). World J Oncol..

[15] Balasubramaniam SD (2019). Med..

[16] Song D (2015). Oncol Lett..

[17] Orbegoso C (2018). Rep Pract Oncol Radiother..

[18] Janicek M, Averette H (2001). Laboratory Medicine..

[19] Menderes G (2016). Expert Rev Anticancer Ther..

